# Distal Femur Megaprostheses in Orthopedic Oncology: Evaluation of a Standardized Post-Operative Rehabilitation Protocol

**DOI:** 10.3390/healthcare11222984

**Published:** 2023-11-19

**Authors:** Lorenzo Andreani, Edoardo Ipponi, Federico Falcinelli, Sara Barderi, Lorenzo Vannucci, Francesco Rosario Campo, Antonio D’Arienzo, Paolo Domenico Parchi

**Affiliations:** 1 Department of Orthopedics and Trauma Surgery, University of Pisa, 56126 Pisa, Italy; lorenzo.andreani.unipi@gmail.com (L.A.); f.falcinelli@studenti.unipi.it (F.F.); s.barderi@studenti.unipi.it (S.B.); francesco.campo@ao-pisa.toscana.it (F.R.C.); antu84@gmail.com (A.D.); paolo.parchi@unipi.it (P.D.P.); 2 Department of Rehabilitation and Physical Therapy, University of Pisa, 56126 Pisa, Italy; lorenzo.vannucci@ao-pisa.toscana.it

**Keywords:** megaprosthesis, orthopedic oncology, functionality, rehabilitation, physical therapy

## Abstract

Background and Objectives: Megaprostheses are the most used reconstructive approach for patients who have undergone massive resection of their distal femurs due to bone tumors. Although the literature about their outcomes has flourished in recent decades, to date, a consensus on rehabilitative treatment is yet to be established. In this study, we report on our experience with our latest standardized rehabilitation program, evaluating our results in a mid-to-long-term scenario. Materials and Methods: We evaluated the functional results of all our oncologic patients treated between 2016 and 2022 who could follow our standardized post-operative rehabilitative approach, consisting of progressive knee mobilization and early weight-bearing. Results: Sixteen cases were included in our study. The average duration of the patients’ hospitalization was 12.2 days. A standing position was reached on average 4.1 days after surgery, while assisted walking was started 4.5 days after surgery. After a mean post-operative follow-up of 46.7 months, our patients’ mean MSTS score was 23.2 (10–30). Our data suggest that the sooner patients could achieve a standing position (R = −0.609; *p* = 0.012) and start walking (R = −0.623; *p* = 0.010), the better their final functional outcomes regarding their MSTS scores. Conclusions: Rehabilitation should be considered a pivotal factor in decreeing the success of distal femur megaprosthetic implants in long-surviving oncologic patients. Correct rehabilitation, focused on early mobilization and progressive weight-bearing, is crucial to maximizing the post-operative functional outcomes of these patients.

## 1. Introduction

Megaprostheses are modular endoprosthetic implants that substitute large bone segments, including metaphyses and shafts, after massive resections in cases with bone tumors, complex fractures, or arthroplasty failures [1]. In particular, years of research and innovations have shaped these prostheses to restore the functionality of patients who have suffered massive bone and soft tissue losses, such as the ones that result from the radical resections of bone tumors [2]. Although prosthetic reconstructions for long bones have been described in literature since the last decade of the 19th century, their use has increased significantly since the 1970s and has been expanding ever since [3,4]. The first megaprosthetic implants introduced in orthopedic oncology were monoblock customized prostheses, which required clinicians to predict the extent of osteotomy based on preoperative imaging data. Then, with the cooperation of engineers, a prosthesis matching the size of the patient’s bone defect and medullary cavity could be manufactured. This high grade of customization was undoubtedly time-consuming and expensive. In particular, weeks and even months of waiting could allow sufficient time for local and systemic spread of the disease. Moreover, the eventual spread of the disease was particularly tough to treat intra-operatively, considering that the unitary design of these implants did not allow for significant intra-operative modifications of the planned surgery. These limitations were overcome with the introduction of modern modular megaprostheses. These ready-to-use implants did not require long design and production times, cutting the waiting time for surgical interventions.

As the extent of osteotomy varies among patients with bone tumors, modular components of different sizes allow a high degree of adaptability, which was previously unmatched among custom-made implants. Furthermore, having standardized implant components and common toolkits increased the level of standardization and reproducibility for these surgical procedures, increasing the experience and the confidence of the most experienced orthopedic oncologists. For all these reasons, modular megaprostheses are still the most used reconstructive approaches for reconstruing the distal femur after bone tumor resections [3,4,5].

Despite the usual sacrifice of tendons, muscles, and knee ligaments [6] and some inevitable changes in patients’ gait, such as shortening of the stance phase for the treated limb and a more prolonged stance phase for the contralateral one [7], these implants are designed to ensure immediate stability and a certain degree of mobility. 

Compared to other reconstructive approaches, such as massive allografts and autografts, megaprostheses can allow for early weight-bearing and quick mobilization of the treated knee after surgery. In this scenario, proper rehabilitation represents an integral part of the therapeutic pathway for oncologic patients treated with distal femur resection and the implantation of megaprostheses.

Maintaining good muscular tone and tropism in the months and years that follow surgery might play a pivotal role in maximizing the functional performance of these implants [8]. Surgical resections of the distal femur in orthopedic oncology can be associated with sacrificing extensive segments of the surrounding soft tissues. Correct physical therapy could counterbalance the muscular deficits that could emerge, sacrificing extensive segments of the surrounding soft tissues. Conversely, prolonged immobilization could increase the risk of developing massive fibrosis and periprosthetic adherences, which, in turn, could limit the effectiveness of the treatment as a whole. For this reason, prosthetic implants, including megaprostheses, generally benefit from progressive mobilization and correct post-operative physical therapy [9,10].

Although the literature has abundant notes about surgical techniques in cases with massive distal femur resection and mega-prosthetic reconstructions, and much has been reported in terms of implants’ complications and post-operative performances [9,10,11], less attention has been paid to the rehabilitation protocols necessary to restore patients’ functionality. While the pivotal role of post-operative rehabilitation in total knee arthroplasty has been extensively highlighted in the recent literature [12,13,14,15], previous studies on distal femur megaprostheses gave little or no data about the rehabilitation programs used [9,10,11]. Detailed descriptions of post-operative rehabilitation protocols have been provided mainly by case reports [16,17]. At the same time, large-sized studies generally did not focus on the post-operative management of the treated limb. The lack of consensus on rehabilitation protocols also reflects the paucity of evidence on post-operative physical treatments for distal femur megaprostheses. 

In this study, we report on our experience with our latest standardized rehabilitation program, evaluating our results in a mid-to-long-term scenario.

## 2. Materials and Methods

This single-center retrospective study was performed following the ethical standards of the 1964 Declaration of Helsinki and its later amendments [18].

Our study consisted of a review of all the oncologic patients treated in our institution with massive bone resection of the distal femur and mega-prosthetic reconstruction of the distal femur and the knee joint between June 2016 and June 2022. 

The inclusion criteria were (I) a massive bone resection followed by the implantation of a modular megaprosthesis of the distal femur and knee joint, (II) a diagnosis of a primary or secondary bone tumor, and (III) the use of our standard rehabilitation protocol during and after each patients’ hospitalization. 

The exclusion criteria were (1) a pre-operative diagnosis other than a bone tumor, (2) pre-operative neurological deficits, adverse effects of chemotherapy, or other systemic diseases that could have impeded the execution of a proper rehabilitation as scheduled in our protocol, (3) the intra-operative sacrifice of the extensor apparatus as a whole in order to achieve wide resection margins, (4) the occurrence of post-operative mechanical failures or local recurrences that required further surgical interventions and thereby compromised patients’ post-operative recovery, and (5) a follow-up shorter than 12 months.

Pre-operative X-rays, CT scans, and MRI images were evaluated before surgery and were used in order to both orient the pre-operative diagnostic process and to aid in surgical planning. Patients’ pre-operative functionality was assessed using the MSTS score.

**Figure 1:**
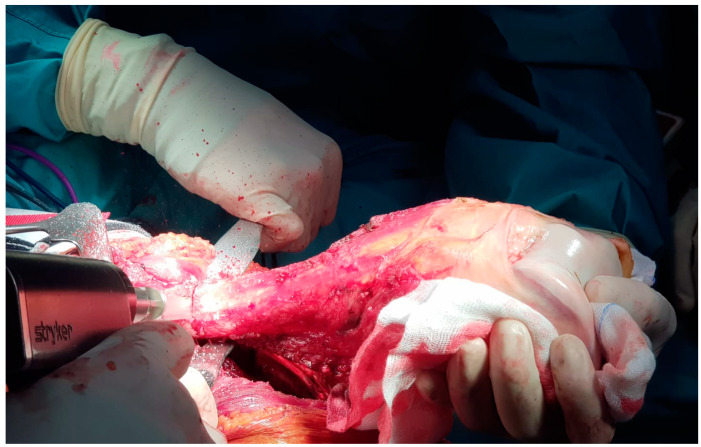
Intra-operative image of a distal femur resection. The distal end of the femur was isolated from the surrounding soft tissues, with the contextual sacrifice of insertion points for tendons and ligaments. The osteotomy line was identified using X-ray guidance and according to pre-operative planning. The bone shaft was exposed using Homan levers and cut using a motorized saw knife.

Once in the surgical theater, patients were set in a supine position. A sterile field covered the thigh, the knee, and the leg of the affected lower limb. The intervention used an antero-lateral approach to the distal femur and the knee. The distal femur was then isolated from the surrounding healthy tissues. An en-bloc resection of the distal femur was carried out for each case. Osteotomy was performed 2 cm away from the tumor’s edge, identifying matching pre-operative MRI images and intra-operative X-rays (Figure 1).

After the osteotomy, the distal femur was resected and sent to our pathologists to confirm the diagnosis of schwannoma using routine histology, histochemistry, and immunohistochemistry techniques. Intra-operative histological evaluations were also conducted on soft tissue samples around the resected area. In case one or more of these samples was involved in the neoplasm, the resection was extended in the same areas until the margins were proven to be free of disease.

The same intra-operative histological evaluations were carried out on the remaining intramedullary canal at the proximal edge of the osteotomy line. If the tumor affected the examined tissue, we performed a further recut of 2 cm, and the procedure was repeated until the histological examination could exclude a neoplastic colonization of the remaining femur shaft. The length of the resected bone segment was recorded intra-operatively for each case.

The megaprosthetic implant of choice was the Distal Femur Megasystem C (Waldemar LINK^®^ GmbH & Co. KG, Hamburg, Germany). We performed intra-operative evaluations of the implants’ stability and mobility throughout the procedure. The length of the treated limb was also assessed and compared to the contralateral one. Patients suffering from primary bone tumors received uncemented implants, whereas cemented stems were used for patients with metastatic diseases. The remaining soft tissues were then sutured to recreate continuity of the muscular and fascial layers and provide a satisfying patella alignment. Two surgical drains were placed before wound closure and removed once the drainage had stopped or had become less than 25 mL/day. Stitches were used to anchor the drains to the patients’ skin, avoiding premature removal of the drains and preventing them from being accidentally pulled out during the first days of rehabilitation. All patients received the same systemic antibiotic therapy. Vancomycin 1 g and Tobramycin 100 mg were administered intravenously every 12 h from the night before surgery until the complete removal of surgical drains. Eventual intra-operative complications were reported. Immediately after surgery, treated knees were set in articulated knee braces with free flexion and extension. We used knee braces to prevent the implant from being the only structure to support varus and valgus stresses, thereby providing even more stabilization for the healing soft tissues and reducing the risk of mechanical loosening of the implants themselves. Braces were maintained full-time and during physiotherapy for 30 days after surgery.

All the patients included in our study carried out their post-operative rehabilitation according to our standardized protocol, described in detail in Table 1.

Our protocol was designed to recover patients’ walking and everyday life activities quickly. Once patients’ clinical conditions were stable and their vital parameters allowed for safe mobilization, our patients received daily physical therapy under the supervision of our physiotherapy and rehabilitation unit. Using cemented or uncemented stems did not determine any change in our protocol.

Since the first day of clinical stability, our physiotherapists assisted our patients in achieving postural transitions in bed and transferring to a sitting position, at first at the edge of the bed, and then, in an armchair. This first step would prepare them for later transitioning to a standing position for walking re-education. The recovery and maintenance of muscle tone and tropism and the knee’s range of motion were encouraged with active and passive foot, ankle, and knee mobilization exercises. Patients were encouraged to perform daily exercises in an isometric contraction of the quadriceps, adductors, glutes, and gastrocnemius, and open-kinetic-chain exercises of the lower limb. The rehabilitation process of each case was based on our standardized protocol, although a certain grade of customization was allowed for each case. For those who could stand our progressive workload, the number of series increased throughout hospitalization. On the contrary, if the patient experienced excessive pain and fatigue, the physiotherapist decreased the workload and the number of series.

**Figure 2:**
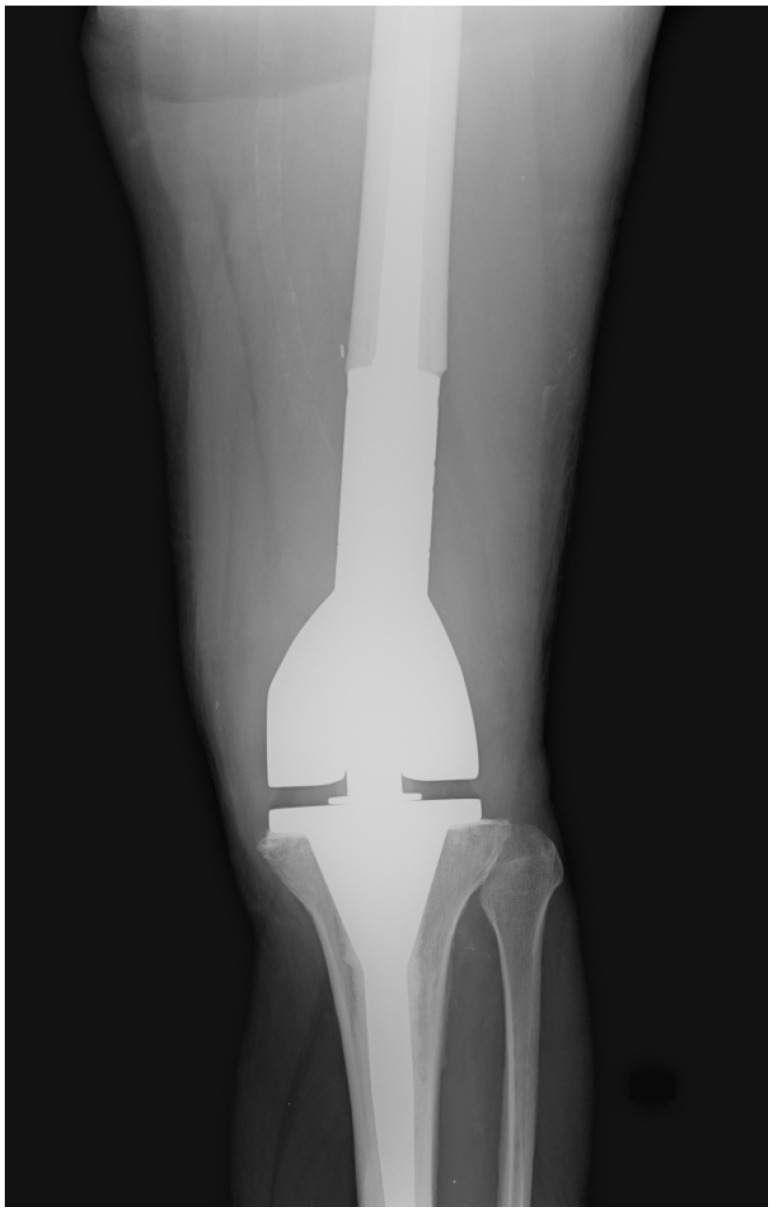
Post-operative X-ray of one of our patients treated with the implantation of a distal femur megaprosthesis.

The post-operative follow-up consisted of serial office visits, clinical evaluations, and X-ray images to assess the surgical treatment’s clinical and radiological outcomes (Figure 2). 

Each post-operative complication (Grade III or higher according to the Clavien–Dindo Classification) that did not represent an exclusion criterion is reported. Complications were divided according to the Henderson failure mode. Each patient’s post-operative functional status was evaluated according to the MSTS score at his or her latest clinical evaluation. 

Statistical analysis was performed using Stata SE 13 (StataCorp LLC, College Station, TX, USA). Statistical significance was set at 0.05 for all endpoints.

## 3. Results

Between June 2016 and June 2022, thirty-two patients were treated with massive resection of their distal femurs and the implantation of a megaprosthesis in our institution. Sixteen received the treatment due to a bone tumor, meeting our inclusion criteria, and were therefore included in our study. Their mean age was 44.1 (15–79).

Among our sixteen cases, fourteen patients suffered from primary bone tumors: eight patients were diagnosed with osteosarcoma, five had chondrosarcoma, and one had a giant cell tumor of the bone. The remaining two patients were suffering from metastatic carcinomas. None of them had pre-operative pathological fractures. On average, their pre-operative MSTS score was 10.4 (5–15).

On average, the resected segment of the proximal femur had a length of 13.6 cm (10–20). None of our patients suffered from major intra-operative complications. All the primary lesions were removed with wide margins of resection.

Among our cases, the mean hospital stay amounted to 12.2 days (2–22). During those days, each patient received active rehabilitative training from our physiotherapists. Our patients could reach a standing position on average within 4.1 (1–7) days after surgery, while assisted walking started within an average of 4.5 (1–10) days after surgery. Crutches were the first walking aids for 68.7% (11) of our patients, while the remaining 31.3% (5) resorted to walking frames. 

Our patients’ mean post-operative follow-up was 46.7 (15–82) months.

Two of our patients suffered from major post-operative complications that did not represent an exclusion criterion for the current study. One patient had a fall 20 days after surgery and had a lacerated and contused wound in her anterior knee. The wound was not in direct contact with the surgical one and did not lead to exposure of the prosthetic implant. It was successfully treated with a suture and oral antibiotic therapy. Another patient had a wound dehiscence, successfully treated with surgical debridement, antibiotic therapy, and negative-pressure wound therapy (NPWT).

At their latest follow-ups, our patients’ mean MSTS score was 23.2 (12–30). 

A schematic summary of our population is reported in Table 2.

According to a Pearson correlation test, there was a direct linear correlation between patients’ age and the duration of their hospitalization (R = 0.623; *p* = 0.010). However, in our cohort, age was not significantly correlated with the number of days necessary to achieve a standing position or to start walking, nor with patients’ functional outcomes, calculated using the MSTS score. No statistically significant correlation was found between resection length and patients’ post-operative MSTS scores. Other Pearson correlation tests demonstrated that the sooner patients could achieve a standing position (R = −0.609; *p* = 0.012) and start walking (R = −0.623; *p* = 0.010), the better their final functional outcomes regarding their MSTS scores.

## 4. Discussion

To date, megaprosthetic implants are among the most suitable options for reconstructing the knee and the distal femur after the resection of bone tumors [6].

Several authors have reported good post-operative functional results after the implantation of distal femur megaprostheses, with reasonable complication rates considering the magnitude of the intervention [3,6,7,9,10,11]. Encouraging functional outcomes have also been reported with broad megaprosthetic reconstruction using implants that replace a large portion of the femoral length [6,19].

Although the modern literature has an abundance of articles on the survival and the functional results of distal femur megaprostheses, little has been written about their functional recovery, and a standardized-consensus rehabilitation protocol is far from being established. Despite the absence of large-scale studies describing post-operative protocols in terms of rehabilitation and physical therapies for megaprostheses, it is reasonable to state that correct mobilization could maximize the post-operative functionality of patients’ knees in the weeks and months that follow surgical treatment. The modern literature has primarily highlighted the importance of proper and rapid recovery protocols for patients who have undergone total knee arthroplasty (TKA), emphasizing the ideas of early mobilization and progressive weight-bearing on the treated limb [12,13,14,15]. TKAs and distal femur megaprostheses differ, as the latter are used for patients who undergo resection not only of the articular surfaces but also of the distal metaphysis and, eventually, a segment of the femoral shaft. The sacrifice of extended bone segments implies the loss of anchoring sites for tendons and ligaments, which can be only partially reconstructed. Despite these differences, some of the principles that guide post-operative clinical practice in total knee arthroplasty should be translated to patients who have received a distal femur megaprosthesis, as it combines a TKA with an extended resection and reconstruction of the distal femur. Especially in oncologic cases, the massive involvement of the soft tissues of the distal thigh and the sacrifice of tendons and ligaments in the knee region can be necessary to resect the tumor with wide margins [6]. The inevitable surgical damage to muscles, tendons, and ligaments should be considered while planning patients’ post-operative rehabilitation. In the days and weeks following surgery, physicians should ensure good stability in the knee, minimizing varus and valgus stresses and avoiding dislocations while walking and during physical therapies [20].

On the other hand, weight-bearing and knee articulation should not be avoided entirely since early mobilization and assisted active walking are necessary to prevent adhesions and consequential dramatic limitations in patients’ knee functionality [21,22]. Considering the necessity to dovetail adequate post-operative stabilization with early mobilization, we carried out early and intense physical treatments. We encouraged our patients to undergo progressive weight-bearing while wearing an articulated knee brace.

Although the general clinical conditions of many oncologic patients might not allow for intense mobilization from the first day after surgery, orthopedic surgeons should be aware of the importance of starting physical treatments as soon as possible.

Our population confirmed the importance of early and intense rehabilitation in order to maximize the post-operative functionality of the treated lower limb. This idea is confirmed by the fact that our cases highlighted statistically significant linear correlations between the timing of standing and walking and patients’ post-operative MSTS scores. Indeed, the sooner our patients could achieve a standing position and start walking, the better their final functional outcomes at their latest follow-up. 

We acknowledge that our study has some limitations. One of them is represented by the retrospective nature of our study, which did not allow for the complete standardization of the post-operative follow-up procedures for each patient. The small size of our cohort represents another limitation. The rarity of these tumors and the limited investigation period did not allow us to operate on a broader population. This partially limited the statistical significance of some of the data associations we wanted to investigate at the beginning of our research. Both of these issues could be overcome in the future by performing similar evaluations on a prospective basis and broader populations, eventually with multicentric studies, which could confirm or refute our findings. 

Beyond these limitations, our study provides an unprecedented focus on the post-operative physical therapies carried out in a referral center after massive tumor resections of the distal femur and the implantation of megaprostheses. Unlike previous studies, whose primary focus was on surgical procedures, patients’ survival, and the evaluation of post-operative complication rates, our study has at its center the mutual correlation between surgical procedures, the first post-operative rehabilitation, and patients’ functional outcomes. To our knowledge, detailed post-operative rehabilitation protocols and post-operative data, such as the timing of standing and walking, had never been extensively considered in previous studies on distal femur megaprostheses. As modern orthopedics is recognizing ever more the importance of correct rehabilitation to maximize the effectiveness of arthroplasty and other surgeries that involve articular segments [12,13,14,15,16], the same mindset should be translated to orthopedic oncology and megaprosthetic surgery.

Our outcomes suggest that proper rehabilitation, focused on early mobilization and progressive weight-bearing, is crucial to maximizing patients’ functional outcomes in mid- and long-term scenarios. As soon as patients reach clinical stability, they should be assisted in postural transitions in bed and transferred to a sitting position. In the following days, patients should be abetted to maintain muscle tone and tropism with active and passive foot, ankle, and knee mobilization exercises. Daily exercises in an isometric contraction of the quadriceps, adductors, glutes, and gastrocnemius and open-kinetic-chain exercises of the lower limb should also be taught and encouraged, preparing patients and easing the transition to the standing position for walking re-education.

Physiotherapists and rehabilitators, inside and outside referral centers, should indeed be instructed to provide daily treatment for those who have been treated with megaprostheses. Although their care could slightly change depending on the necessities of every case, its intensity should never be limited for fear of harm, nor should it be limited to the first weeks following the surgical treatment. 

Rehabilitation should be considered a pivotal factor in decreeing the success of megaprosthetic implants in long-surviving patients, as good post-operative performance could bring them back to their activities of daily living and lead to consistent improvement in their quality of life.

## 5. Conclusions

In conclusion, rehabilitation is essential to the treatment pathway of patients who have received megaprosthetic implants due to distal femur bone tumors. Although physical therapies should be customized for each patient, a standardized protocol focused on early knee mobilization and weight-bearing should be recommended to maximize patients’ post-operative functional recovery in the weeks and months that follow the surgical intervention. Larger studies with a broad focus on the rehabilitation of distal femur megaprostheses, even with different rehabilitative protocols, would be beneficial to increase and broaden physicians’ and rehabilitators’ knowledge on this topic.

## Figures and Tables

**Table 1:** Rehabilitation stages: distal femoral replacement with megaprosthesis.

	First Week after Surgery	Second Week after Surgery	Third–Fourth Week after Surgery	After the First Month after Surgery
Weight-bearing on operated leg	Partial	Progressive partial	Full	Full
Restoration and maintenance of muscle tone and trophism	+	++	++	+++
Restoration and maintenance of joint motion	++	++	++	+++
Postural passages	+++	+++	++	+
Gait re-education	+++	+++	+++	++
Stair climbing re-education	+	++	++	+++
Proprioceptive exercises	+	++	++	+++
Education about hygienic and behavioral rules	+++	+++	+++	+++

+: Low intensity activity; ++: Mild intensity activity; +++: High intensity activity.

**Table 2:** A summary of the outcomes in our patients.

Case	Age	MSTS Pre	Diagnosis	Enneking Classification	Resection (mm)	Complications	Hospital. (Days)	Standing (Day)	Walking (Day)	MSTS Post	F-U (Months)
1	69	11	MTS Carcinoma	III	100	No	17	5	7	24	15
2	53	10	Chondrosarcoma	IIB	175	No	7	1	1	27	17
3	15	10	Osteosarcoma	IIA	100	No	10	5	6	18	20
4	46	5	Chondrosarcoma	IIB	140	No	9	3	3	18	20
5	19	15	Osteosarcoma	IIA	150	No	9	2	3	30	31
6	79	8	Chondrosarcoma	IIB	160	No	16	2	2	24	32
7	71	12	Osteosarcoma	IIA	110	Fall and LC wound	16	4	5	16	44
8	47	13	Osteosarcoma	IIB	130	No	9	2	2	30	46
9	61	5	MTS Carcinoma	III	160	Dehiscence	22	10	10	12	47
10	44	14	Chondrosarcoma	IB	95	No	19	9	9	24	54
11	48	14	Bone GCT	IA	120	No	11	5	5	25	60
12	44	12	Osteosarcoma	IIA	130	No	12	6	6	26	64
13	19	13	Osteosarcoma	IIA	120	No	9	4	4	24	68
14	34	7	Osteosarcoma	IIB	160	No	9	1	1	27	70
15	16	11	Osteosarcoma	IIA	130	No	8	1	1	28	78
16	40	6	Chondrosarcoma	IIA	100	No	12	6	7	18	82

MTS = metastasis; Hospital. = hospitalization days; Standing = standing position achieved X days after surgery; Walking = assisted walking started X days after surgery; F-U = follow-up.

## Data Availability

The data that support the findings of this study are available from the corresponding author upon reasonable request.
